# How to deliver an oral presentation

**DOI:** 10.1097/IJ9.0000000000000025

**Published:** 2017-06-08

**Authors:** Georgina Wellstead, Katharine Whitehurst, Buket Gundogan, Riaz Agha

**Affiliations:** a Lister Hospital, East and North Hertfordshire NHS Trust; b Royal Devon and Exeter Hospital; c University College London; d Guy's St Thomas' NHS Foundation Trust, London, UK

**Keywords:** How to, Presentation, Conference, Oral Presentation

## Abstract

Delivering an oral presentation in conferences and meetings can seem daunting. However, if delivered effectively, it can be an invaluable opportunity to showcase your work in front of peers as well as receive feedback on your project. In this “How to” article, we demonstrate how one can plan and successfully deliver an engaging oral presentation.

Giving an oral presentation at a scientific conference is an almost inevitable task at some point during your medical career. The prospect of presenting your original work to colleagues and peers, however, may be intimidating, and it can be difficult to know how to approach it. Nonetheless, it is important to remember that although daunting, an oral presentation is one of the best ways to get your work out there, and so should be looked upon as an exciting and invaluable opportunity.

## Slide content

Although things may vary slightly depending on the type of research you are presenting, the typical structure is as follows:

- Opening slide (title of study, authors, institutions, and date)
- Background
- Study aims
- Methodology
- Results
- Discussion (including strengths and weaknesses of the study)
- Conclusions
- References

Picking out only the most important findings to include in your presentation is key and will keep it concise and easy to follow. This in turn will keep your viewers engaged, and more likely to understand and remember your presentation.

Psychological analysis of PowerPoint presentations, finds that 8 psychological principles are often violated^1^. One of these was the limited capacity of working memory, which can hold 4 units of information at any 1 time in most circumstances. Hence, too many points or concepts on a slide could be detrimental to the presenter’s desire to give information.

You can also help keep your audience engaged with images, which you can talk around, rather than lots of text. Video can also be useful, for example, a surgical procedure. However, be warned that IT can let you down when you need it most and you need to have a backup plan if the video fails. It’s worth coming to the venue early and testing it and resolving issues beforehand with the AV support staff if speaking at a conference.

## Slide design and layout

It is important not to clutter your slides with too much text or too many pictures. An easy way to do this is by using the 5×5 rule. This means using no more than 5 bullet points per slide, with no more than 5 words per bullet point. It is also good to break up the text-heavy slides with ones including diagrams or graphs. This can also help to convey your results in a more visual and easy-to-understand way.

It is best to keep the slide design simple, as busy backgrounds and loud color schemes are distracting. Ensure that you use a uniform font and stick to the same color scheme throughout. As a general rule, a light-colored background with dark-colored text is easier to read than light-colored text on a dark-colored background. If you can use an image instead of text, this is even better.

A systematic review study of expert opinion papers demonstrates several key recommendations on how to effectively deliver medical research presentations^2^. These include:

- Keeping your slides simple
- Knowing your audience (pitching to the right level)
- Making eye contact
- Rehearsing the presentation
- Do not read from the slides
- Limiting the number of lines per slide
- Sticking to the allotted time

## Rehearsal

You should practice your presentation before the conference, making sure that you stick to the allocated time given to you. Oral presentations are usually short (around 8–10 min maximum), and it is, therefore, easy to go under or over time if you have not rehearsed. Aiming to spend around 1 minute per slide is usually a good guide. It is useful to present to your colleagues and seniors, allowing them to ask you questions afterwards so that you can be prepared for the sort of questions you may get asked at the conference. Knowing your research inside out and reading around the subject is advisable, as there may be experts watching you at the conference with more challenging questions! Make sure you re-read your paper the day before, or on the day of the conference to refresh your memory.

## Handouts

It is useful to bring along handouts of your presentation for those who may be interested. Rather than printing out miniature versions of your power point slides, it is better to condense your findings into a brief word document. Not only will this be easier to read, but you will also save a lot of paper by doing this!

## Delivering the presentation

Having rehearsed your presentation beforehand, the most important thing to do when you get to the conference is to keep calm and be confident. Remember that you know your own research better than anyone else in the room! Be sure to take some deep breaths and speak at an appropriate pace and volume, making good eye contact with your viewers. If there is a microphone, don’t keep turning away from it as the audience will get frustrated if your voice keeps cutting in and out. Gesturing and using pointers when appropriate can be a really useful tool, and will enable you to emphasize your important findings.

## Presenting tips

- Do not hide behind the computer. Come out to the center or side and present there.
- Maintain eye contact with the audience, especially the judges.
- Remember to pause every so often.
- Don’t clutter your presentation with verbal noise such as “umm,” “like,” or “so.” You will look more slick if you avoid this.
- Rhetorical questions once in a while can be useful in maintaining the audience’s attention.

## Finishing

When reaching the end of your presentation, you should slow down in order to clearly convey your key points. Using phases such as “in summary” and “to conclude” often prompts those who have drifted off slightly during your presentation start paying attention again, so it is a critical time to make sure that your work is understood and remembered. Leaving up your conclusions/summary slide for a short while after stopping speaking will give the audience time to digest the information. Conclude by acknowledging any fellow authors or assistants before thanking the audience for their attention and inviting any questions (as long as you have left sufficient time).

If asked a question, firstly thank the audience member, then repeat what they have asked to the rest of the listeners in case they didn’t hear the first time. Keep your answers short and succinct, and if unsure say that the questioner has raised a good point and that you will have to look into it further. Having someone else in the audience write down the question is useful for this.

## Conclusions

The key points to remember when preparing for an oral presentation are:

Keep your slides simple and concise using the 5×5 rule and images.
When appropriate; rehearse timings; prepare answers to questions; speak slowly and use gestures/ pointers where appropriate; make eye contact with the audience; emphasize your key points at the end; make acknowledgments and thank the audience; invite questions and be confident but not arrogant.
